# Client Views of Contingency Management in Gambling Treatment: A Thematic Analysis

**DOI:** 10.3390/ijerph192417101

**Published:** 2022-12-19

**Authors:** Lucy Dorey, Jack McGarrigle, Richard May, Alice E. Hoon, Simon Dymond

**Affiliations:** 1 School of Psychology, Swansea University, Singleton Campus, Swansea SA2 8PP, UK; 2 School of Psychology and Therapeutic Studies, University of South Wales, Pontypridd CF37 1DL, UK; 3 Swansea University Medical School, Singleton Campus, Swansea SA2 8PP, UK; 4 Department of Psychology, Reykjavík University, Menntavegur 1, Nauthólsvík, 101 Reykjavík, Iceland

**Keywords:** contingency management, gambling, treatment, thematic analysis, qualitative

## Abstract

Low levels of treatment access and poor retention among those with gambling problems suggests a need to improve treatment. Contingency management (CM) is a behavioural intervention involving the identification of target behaviours and the provision of incentives when targets are met. There exists a substantial evidence base for CM increasing abstinence and attendance in substance misuse treatment, but this has not been widely extended to gambling treatment setting. This study sought to explore the views of clients about CM for the treatment of problematic and disordered gambling. We conducted semi-structured interviews with 25 gambling treatment clients who were, or had previously been, engaged in treatment in Great Britain. Participants were provided with an explanation of CM, two hypothetical scenarios, and two structured questionnaires to facilitate discussion. Thematic analysis was used to interpret findings. Some participants felt that clients could manipulate CM while in treatment to obtain money to gamble, and that mechanisms of CM could trigger recovering clients into relapse. Participants also identified potential benefits of CM to achieve treatment goals, by enhancing motivation and engagement while in treatment, and helping bring people into treatment earlier. Gambling treatment clients broadly supported the use of incentives for treatment. CM is seen as a facilitator of extended engagement in treatment, and an encouragement for clients to make progress in the treatment process.

## 1. Introduction

Gambling is a growing international public health issue that can have negative, harmful consequences across several domains [1]. Treating difficulties that arise due to harmful gambling requires effective, evidence-based intervention, yet most individuals do not seek treatment and a large proportion drop out before completion [2]. For example, in 2019, 9008 people in Great Britain accessed treatment from the National Gambling Treatment Service, which is less than 5% of adults who might benefit [3]. Consequently, more research is needed into approaches that could increase gambling treatment attendance and reduce drop-out rates.

Contingency management (CM) is a behavioural intervention with high efficacy at reducing addictive behaviours, increasing adaptive behaviours and treatment attendance [4,5,6,7,8]. Based on principles of operant conditioning, CM involves the identification of target behaviours (such as attendance at therapy, abstinence from addictive substances, or completing steps towards one’s recovery goals, etc.) and the provision of tangible reinforcers (such as vouchers or credits towards the purchase of preferred items) when evidence of the target behaviour is produced [4,9]. According to principles of positive reinforcement, a target behaviour should increase in frequency when it is followed by the reinforcer, helping to foster new behaviour. CM has been successfully applied in areas such as alcohol and substance-use disorders, medication adherence, and other health-related behaviours [10,11,12,13].

CM is a robust methodology. CM approaches are highly manualized, and follow pre-described protocols for assessing, implementing, and recording attendance and abstinence [5,6,7,8,9,10]. These procedures are managed by professionals, are typically checked by others for fidelity, and use observable evidence (e.g., biological and behavioural measures) that provide demonstrative validity. The focus on observable measures elicits clear evidentiary designations. For example, evaluations of CM provider competence tools from 1613 audiotapes of therapist interactions with 78 patients enrolled in the training and 103 patients in the randomized phases found good to excellent interrater reliability (ICC 0.92) and internal consistency (Cronbach’s alpha 0.834–0.903), with a significant correlation with therapeutic alliance (*r*^2^ = 0.36, *p* < 0.001), and were predictive of longest continuous abstinence (*F*(1, 99) = 3.83, *p* < 0.05) [14]. This is in contrast to widely used gambling assessments used in the UK and world-wide like the Problem Gambling Severity Index (and other measures) that typically fail to capture the complexity of gambling or reliably measure gambling behaviour [15].

Moreover, systematic reviews and meta-analyses of CM have found CM leads to larger treatment effect sizes, increases the likelihood of abstinence, and greater attendance rates compared to other addiction treatment approaches [4,8,13]. For example, CM has been shown to increase treatment attendance in substance-use treatment programs and vaccination-sessions for individuals with complex needs [7,8,13]. Recently, a systematic review and meta-analysis of 74 randomized clinical trials and 10,444 opioid dependent adults found CM increase abstinence rates for substance use (psychomotor stimulants, polysubstance use, illicit opioids, and cigarettes) and improved treatment attendance and medication adherence [16].

Despite the supportive evidence base for adding CM to substance misuse treatment, implementation within the field of gambling treatment has been slow [12]. Given the range of treatment approaches that exist for treating gambling related problems (e.g., cognitive behavioural therapy, motivational interviewing, pharmacotherapy), CM confers several advantages such as being a positive procedure promoting behaviour change consistent with recovery from gambling, and that it may be used an adjunct to existing treatment(s). Investigations into provider views of CM have found a diversity of opinions, with some supporting incentives while others reporting strong philosophical and practice disagreements, and others highlighting the financial issues implementing CM for gambling disorder [17].

Investigations of client perspectives have found high acceptability of CM to clients receiving treatment for smoking, cocaine addiction, and alcohol consumption [18,19,20,21,22,23,24,25,26,27,28]. Recently, Getty et al. [29] adapted provider questionnaires to produce the *Service User Survey of Incentives* (SUSI). London based service users (N = 181), most of whom had no prior experience of CM (92.3%), completed the SUSI. It was found that overall, 81% of patients were in favour of the use of CM interventions in UK substance misuse services. Clients endorsed CM that targets attendance (72.4%), the reduction of unhealthy behaviours (72.9%), and increasing healthy behaviours (81.7%). Negative items were generally less strongly endorsed, but some clients’ responses showed they had concerns about the implementation of CM (e.g., 32% endorsed the belief that most clients would sell or exchange incentives to find money for substances, and 28% agreed that incentives could interfere with clients developing ‘internal motivation’). Getty et al. discussed the need for qualitative research questions to explore the reasoning and motivations for beliefs towards CM and identified scope to explore the extent to which contextual factors may have an influence on clients’ beliefs and views of CM. Previously, Kirby et al. [11,24] found an association between attitudes toward CM and contextual factors of treatment providers, suggesting that an exploration of client characteristics could lead to a more detailed understanding of the potential barriers to the implementation of CM in treatment settings.

CM may hold promise for extending abstinence, increasing attendance and other positive behaviours in treatment for harmful gambling [30,31]. Previous qualitative studies have contributed to understanding of how clients respond to and view CM withing substance misuse services. Moreover, clients’ views of CM also overlap with those of practitioners. Leickly et al. [32] recruited 35 adults with co-occurring serious mental illness and alcohol dependence to semi-structured interviews after taking part in a CM trial targeting abstinence from alcohol. They found that clients were appreciative and enthusiastic about receiving prizes and reported that they felt it helped them to stay abstinent, increased accountability, awareness of their drinking, and increased motivation. Similarly, Neale et al. [33] interviewed 20 substance misuse clients who were receiving supervised injectable opioid treatment alongside CM. While clients mostly felt CM had helped them avoid relapse, and they felt happy and proud to receive the incentives, some were suspicious of the CM intervention, had ethical concerns, or did not have a clear understanding of the rationale and process of the CM. Examining the perceptions of CM using qualitative interviews might provide new evidence for client preparedness and their initial expectations for engaging in CM treatments. Such evidence could better tailor CM for treatment seekers, identify those amenable to CM, and address concerns stopping those from engaging in CM for gambling.

The aim of the present study was to address the research question: “What views do clients of gambling treatment services express when presented with an explanation of CM and examples of how it might apply to gambling?” We also sought to identify contextual influences on clients’ views and to arrive at a nuanced understanding of the factors at play in the potential wider adoption of CM for gambling.

## 2. Materials and Methods

### 2.1. Participants and Design

We used a qualitative approach grounded in a contextual and pragmatic philosophy, using semi-structured interviews. We recruited clients aged over 18 years old who were currently or had been engaged in receipt of gambling treatment services and support in England, Scotland, or Wales (i.e., Great Britain). Within Great Britain, there exists a range of services (e.g., telephone, online, and face-to-face treatment) for individuals (adolescents, adults, gender specific cohorts) and groups offered by multiple agencies. In addition, the UK offers clients seeking gambling support residential, housing, and retreat services. These agencies use a range of assessment tools and procedures, but typically these cover a range of psychiatric issues characterized by ICD-11 and DSM-V taxonomies and are delivered by experienced and nationally certified trained personnel using evidence-based approaches (i.e., cognitive behavioural therapies, relapse prevention, and motivational interviewing), tailored to client needs and their process of recovery. Other support groups include Gamblers Anonymous, a peer-based support group following the alcoholic anonymous model. A convenience sampling approach was used [17]; we originally aimed to recruit 30 clients, but this was revised to 25 as the analysis was not leading to identification of new items of analytic interest (codes) in the data.

Of the 25 clients that participated, the most common forms of gambling were online gambling on casino-like games, sports betting, and fruit/slot machines. The most common types of treatment were Gamblers Anonymous, CBT, and counselling, respectively.

All clients received a £25 Amazon voucher on completion of the study. Ethical approval was received from Wales Research Ethics Committee 5 on 27 January 2021.

### 2.2. Procedure

Gambling treatment services circulated recruitment emails to clients or distributed a flyer about the study during treatment sessions. Social media adverts on Twitter were promoted within the gambling recovery community, and advertisements provided a link to the study website. Potential clients were provided with a Participant Information Sheet and were invited to book a research appointment via an online booking system. Initial phone or videoconferencing meetings were held to confirm informed consent, and a further research meeting was booked.

Interviews and analysis were conducted by the first author. Each client attended one semi-structured interview (and no other persons present) lasting one and a half hours between March–August 2021, which were audio recorded online in Zoom or Teams.

The interview process and topic guide (see Supplementary Materials) were first piloted with individuals with lived experience of harmful gambling. Clients completed an online consent form and background demographics survey hosted in Qualtrics (20 min duration) and were provided with a brief description of CM. Participants were then invited to describe and explore their initial reactions to the approach applied to gambling treatment generally as well more specifically how they might have responded to this approach if this had been an available treatment option. A further question asked participants to identify: “What type of barriers to success do you think this type of approach might encounter?”.

Clients were then asked to identify strengths and weaknesses of two scenarios (adapted from [11,20]). Clients then completed two surveys (10 min): an adapted version of the *Provider Survey of Incentives* (PSI) [11] and *Contingency Management Adoption Attitudes* (CMAA) scales [14], with adjustments made to the gambling treatment setting and removal of items deemed not relevant (see Supplementary Materials). The PSI is a 44-item scale which assesses the attitudes of practitioners regarding the use of CM incentives in addiction treatment settings. Items address potential beliefs and objections to CM such as potential jealousy between clients and people being drawn into treatment for the wrong reasons. Less common concerns included CM being labour intensive CM negatively impacting motivation, the effects of CM not lasting, and CM clashing with the personal philosophy held by practitioners. Additional research has found exposure to CM interventions is associated with more positive provider beliefs [23], and that training workshops can positively impact beliefs [24]. A 35-item version was employed here. The CMAA is a 3-item scale assessing the acceptability of CM for both abstinence and treatment attendance and the potential positive impact it might have on the client-therapist relationship [34].

Clients were invited to comment further on their views after responding to the questions. As many had no prior knowledge of CM, we felt this approach would provide enough information for clients to explore their views.

### 2.3. Approach to Data Analysis

The first author transcribed and checked interview audio data using NVivo Transcription online service. Transcripts were not further commented on by clients. Data were analysed using Reflexive Thematic Analysis (RTA), an approach that captures key aspects of the data repeated by different clients and allows both descriptive and interpretive accounts of the data. RTA is suited for understanding client views of a topic [35,36] and consists of six stages: familiarisation, generating codes, constructing themes, revising themes, defining themes, and producing the report. Reflexive notes recorded during the initial reading of the data (familiarisation phase) highlighted differences between the views of participants about CM for gambling and the literature on practitioner and client views of CM for substance misuse. Thus, complete inductive coding rather than selective deductive coding was used to capture the full range of perspectives in relation to gambling.

A pragmatic contextualist philosophical position places the ‘human act in context’ [37] at the center of scientific enquiry, and asks: what are the contexts that shape the research? Thus, it was important to reflect on three aspects of the research: (1) the contexts that contributed to forming the views of clients, (2) the contexts that shape the articulation of views, and (3) the contexts that shape the interpretation. Prior experiences can influence beliefs and feelings, and these components combine to form the content of an attitude. As clients will not have direct experience of CM for gambling, they would be expected to draw on experiences they relate to the object. This leads to the question: what experiences do clients relate to the process of forming their views of CM for gambling?

The context of the verbalization may influence expressed views in terms of social adjustment, and ego-defensive; to reduce these influences the interviews included space for general conversation to build rapport, non-judgmental attitudes were conveyed whenever the opportunity arose, and positive, negative, and neutral or conflicting views were deliberately encouraged, and treated as equally helpful responses. In relation to the act of interpreting views, knowledge is not separate from the knower (from a contextualist perspective), so the analysis is influenced by the researcher’s viewpoint even when attempts are made to stay close to clients’ intended meaning in the analysis. Reflexivity was therefore important to this analysis. Throughout the analysis reflections on the patterns in the data were recorded.

Complete inductive coding was used to capture all aspects of the data relevant to the research questions. During analysis, a fifteen-point checklist for good Thematic Analysis was followed. The coding tree is provided in Table 1. Coding and initial analysis were carried out by the first author, and a descriptive summary of the data and candidate themes were discussed with the project steering group, which included four researchers and three members with lived experience of harmful gambling, offering another perspective. The analysis brought together interrelated aspects of the data to develop themes from across the codes [38]. Themes were discussed within a steering group including people with lived experience of gambling problems.

## 3. Results

Twenty-five clients were interviewed, and their characteristics are presented in Table 2. The following terms will be used to indicate the numbers of clients in the sample expressing a view: *occasionally* or *uncommonly* indicates three or less; *some* or *several* indicates between four and nine; *many* or *commonly* indicates ten to twelve; *typically* or *often* indicates thirteen to fifteen; *most* indicates sixteen to nineteen; and *almost all* indicates twenty or more.

### Contextual Factors

It was uncommon for clients to have had any experience of incentives in treatment. To form their views, clients drew on various aspects of experience that they related to CM for gambling: other forms of incentives experienced in daily life, their own gambling and recovery experiences, and their experiences of others in recovery.

Four themes ordered according to phases of recovery as described by participants, were constructed from the analysis: (1) dishonesty of active addiction disrupting CM, (2) get people through the door and keep them coming back, (3) Avoidance of triggers in early recovery could be contradicted by CM, and (4) CM could ‘spur you on’ in early recovery. These themes are summarised in Table 3.

*Theme 1: Dishonesty of active addiction disrupting CM*

This theme explores the idea that in the context of active addiction, people are driven to dishonesty by their compulsion to gamble, and that some clients can be expected to manipulate the CM intervention to obtain money to gamble. Several clients highlighted that individuals in the throes of addiction could be out of control, and desperate to gamble. In this context some considered that clients were capable of ‘lip service’, and ‘manipulation’.

Most clients expressed strong concerns about people potentially manipulating a bank statement checking system used to confirm abstinence, as they could use methods of gambling that did not show on their bank statement, e.g., ‘getting paid in cash’, ‘opening multiple bank accounts’. Some saw potential to counter these concerns, for example by asking for receipts for all cash expenditure and for grocery shopping or corroboration from a family member.

It was also considered likely by most clients that gamblers in active addiction could go to great lengths to sell their incentives to obtain money to gamble. It was seen as relatively easy to turn a voucher or purchased item into cash, and that if a person was intent on gambling and needed money, they would be likely to sell the incentive to gamble. Several clients felt that only a small minority would sell incentives. Others suggested ways to counter this, for example some commented that staff organising purchases would be more helpful than receiving vouchers, the incentive could be something that was difficult to sell, or that no-one would buy.

Another aspect of this theme was the commonly expressed view that people with other addictions might fake a gambling addiction to receive incentives, or that people might have a gambling addiction but come only for the incentive without any intention of participating in the treatment process.

Several clients expressed concerns that trust in the therapeutic relationship could be impacted negatively because of the potential for clients to try to manipulate the system. Disagreements could arise over the need to provide evidence of attaining goals, and over what incentives have been earned.

*Theme 2: Get people through the door and keep them coming back*

This theme explores the core idea that CM could help to bring people into treatment earlier and keep them coming back. Some clients described personal experiences of gambling addiction and associated harms lasting many years before successfully engaging in treatment. In this context it was seen as important to try to relieve the suffering and loss associated with ongoing gambling by encouraging people into treatment earlier than might occur naturally.

Clients commonly spoke about the benefits of getting someone through the door and being able to influence them to engage in a process of change, for example through experiencing peers speaking about recovery.

*“Who knows where that could take them, they might hear something, because they’ll be reflective of their own life and maybe reflective of others round about them who’ve actually gone beyond where they are and engaged in life”.*

(P1)

Many clients described witnessing others starting treatment or GA and dropping out and could see potential to keep people coming back to treatment using incentives. They shared their own experience of it taking some time for the message from GA or treatment to sink in: “the more you listen to something, the easier it sinks in” (P5). They considered that the nature of the intervention alongside CM needs to be able to inspire and influence the individual to engage in the treatment or recovery process.

*Theme 3: Avoidance of triggers in early recovery could be contradicted by CM*

This theme explores the idea that avoiding gambling triggers is a key strategy used when setting out on the recovery journey, and concerns that CM could put gamblers in contact with some types of triggers that they would not be able to resist (at this early stage) and could potentially lead to relapse.

*“I want to connect and stop gambling, but then when the urge would come the temptation would come. Yeah. And the temptation to deceive would come back, if I’m desperate”*.

(P17)

Some clients described avoiding carrying money, restricting access to money, or not carrying a phone early in their recovery as strategies to avoid acting on impulses to gamble. In this context, many commented that money should never be used as a reward; “it can’t be because it’s too easy to fall back into temptation again” (P2).

Another type of trigger that were considered best avoided were any activities seen as like gambling, such as building up credits through a loyalty card, raffles and collecting vouchers. Several clients expressed concerns that CM could be like gambling and reinforce gambling related behaviours, such as ‘thinking that whenever you do something you should get rewards’, ‘getting bonuses and perks’, and “not putting the correct effort into something to get something out of it” (P19).

A third iteration of this theme was that feelings of jealousy, failure or unfairness could arise when a person does not achieve their goals, but others do, and these internal triggers could lead to disengagement with treatment or relapse. Some clients suggested that if they did not achieve the incentive a person might gamble to relieve feelings related to failure. Some felt that jealousy is already an issue in gambling recovery groups, and they felt it could also be an issue with CM, because sometimes people are sensitive in recovery and might feel hard done by if they do not get the incentives. Occasionally, clients expressed concerns that reductions in incentives when sessions are missed could be perceived as unfair by those who have genuine reasons why they are unable to attend a session, such as care responsibilities, or having to work late.

*Theme 4: CM could ‘spur you on’ in early recovery*

This theme explores the core idea that early recovery (where the person is now oriented to change) can be full of difficulties and lacking in natural rewards. In this context, incentives were seen as potentially encouraging clients by acknowledging small steps towards change, and through this the incentives could enhance motivation. Furthermore, the types of incentives provided could be related to an individuals’ recovery, as well as target a wide range of recovery goals.

Several clients highlighted how difficult the early stages of recovery can be; people sometimes experience multiple addictions, mental health issues, are facing up to the harm caused by gambling, and “have reminders of gambling everywhere around you” (P15). These clients felt that the rewards that you get from recovery “can be a very long time to notice” (P23), and that in this context, external rewards could be beneficial to achieving abstinence and recovery.

Some clients suggested incentives would be appreciated because they would feel proud and encouraged. Several felt that by identifying and rewarding goals, clients would have small steps recognised and be able to see they are making progress. Where some might see themselves as a failure or not good at anything, it could help them feel that they have achieved something. Occasionally, clients felt that incentives could spur the person on to achieve the same as others they observe making progress; “it could make the other person think, you know what, they’re doing well, I want to do that” (P16).

Incentives were seen as supportive to recovery when they provide a positive experience a client might not otherwise access (e.g., meeting basic needs such as food, to pay a bill, or place credit on a person’s phone; small luxury items or treats; or service-based incentives such as movies, health club, sports coaching, theatre, meals out, spa, music, and days out). Some clients considered that the value of the incentive would mean more to a person in hardship that someone with a good income; “six pound is a tremendous amount of money if you’re destitute through gambling” (P1); “if you’re a banker or whatever, if you’ve got a high paid job, you know, it’s not going to make a lot of difference” (P11). Several clients highlighted the need to make the incentives relevant to the individual’s interests and needs, and to provide choice. Services were seen as beneficial in themselves as they could encourage new hobbies, socialising, reconnecting with people, fill time, and generally take the mind away from gambling-related thoughts.

*“When I was doing something like that, like going to Chester Zoo, it’s more fun for me than gambling, and I get my mind off gambling for a few hours”*.

(P17)

It was also seen by some as beneficial that services could include family members; gamblers are likely to have been disconnected from family when they were preoccupied with gambling and these activities were seen as an opportunity to reconnect.

*“[Because of the gambling] you’ve never given that person personal attention….your mind was always somewhere else. So the restaurant would let them reap the rewards of you not gambling as well… I’m seeing the quality time of being with that person”*.

(P3)

Several clients felt that incentives could be expanded to target recovery related goals other than abstinence and attendance. This could involve identifying what are important life directions for everyone in recovery (relationships, fun/leisure, work, personal growth, mental health, environment, and possessions such as a car), and/or creating a recovery plan.

## 4. Discussion

The present study aimed to explore clients’ views of the potential of CM for gambling treatment. Overall, four key themes characterizing clients’ perceptions of CM were developed from the data. Themes one and three point to potential unintended negative consequences of CM: concerns that some clients in active addiction can be expected to manipulate the CM intervention to obtain money to gamble, and that CM could put recovering gamblers in contact with some triggering situations that would make them vulnerable to relapse. In contrast, themes two and four point to potential benefits of CM for gambling: that CM could help to bring people into treatment earlier and keep them engaged, and that incentives would help clients in active change, by providing recognition of small steps towards change, and enhancing motivation. The themes are somewhat contradictory in nature, and clients often held contradictory views; such ambivalent attitudes are likely to reflect lack of direct experience and knowledge of CM, and people holding these views are likely to be more receptive to changing their views.

Clients differentiated between those in ‘active addiction’, those who were ‘help seeking’ but still unsure about change, or uncertain of how to change, those who were actively changing, but still vulnerable to relapse, and those who had maintained a period of recovery. Their descriptions of the phases of change have similarities to the trans-theoretical model of change [39] which proposes stages of motivation a person moves through to change behaviour: pre-contemplation, contemplation, determination, action, maintenance, and relapse.

When clients are actively changing, incentives were seen as an acceptable way of encouraging and celebrating progress that would improve outcomes. This is in line with the strong positive views expressed by substance misuse clients when they have been exposed to CM [18,19,20,21,22,23,24,25,40,41,42]. The Recovery Model points to a broader range of treatment goals than reduction in abstinence or reducing addictive behaviour: physical, emotional, spiritual, relational, and occupational health domains are considered important targets for change in the recovery model [43,44]. Clients’ views emphasized that incentives could be beneficial if they target broad recovery goals, and the use of incentives themselves can be related to recovery goals (e.g., going for a meal with family). CM interventions linked to the recovery model is a potential area to explore in further research.

In ‘active addiction’ gamblers were portrayed as driven by the compulsive elements of gambling, in line with widely accepted understanding that pathological gamblers do not have the ability to choose to stop their habitual patterns [45]. In this context, it was seen as likely gamblers would obtain money to gamble by whatever means they could, including deception. It is therefore important to be aware of the potential to inadvertently incentivize deceptive behaviour when designing CM interventions for gambling, to minimize opportunities for deception.

Concerns expressed by clients about people attending for the wrong reasons reflects the view that at the stage of active addiction, or pre-contemplation, a person is unlikely to be influenced by treatment, and thus will waste resources. However, in Theme 3 the opposite view was expressed: that some people motivated primarily by incentives could potentially be influenced once attending treatment. Motivational Interviewing, an intervention and style of therapy that aims to facilitate movement from pre-contemplation and contemplation to active change, has strong evidence of effectiveness [46]. This suggests that there is potential to influence clients if engaged through CM. Clients also recognized the importance of shortening duration of harmful active gambling prior to help seeking, and of retaining people in treatment or peer groups; this corresponds with what is known about low rates of treatment seeking and high dropout rates [2,3,13].

Clients’ views suggest they believe that avoidance strategies have an important role in early recovery, and that CM could trigger relapse. Their view is that until the person learns to cope with triggers, avoidance is the best strategy. However, long-term avoidance may not be practical in many situations, and as treatment such as CBT unfolds clients are expected to learn to cope with different types of triggers [46]. Furthermore, we would expect CM to decrease the likelihood of relapse [4,13] rather than increase it. However, some clients might be more vulnerable to triggers than others. This suggests that practitioners delivering CM would need to work with clients to highlight how CM could interact with their personal triggers and address these triggers as part of the treatment process.

## 5. Strengths and Limitations

This study recruited 25 UK based gambling treatment clients, most of whom had a period of abstinence and considered themselves to be in recovery; this context should be taken into consideration when relating these findings to different groups and should not be seen as representative of the wider population of gambling clients. A notable strength of the present findings was the consistency of clients’ views of CM regardless of their prior experience of different treatment approaches (e.g., CBT and Gamblers Anonymous). The background survey of key demographics could have also assessed comorbid alcohol and substance use. The interviews produced in-depth accounts of participant’s views, and sample size was adequate to identify commonly held views, with later interviews producing no new codes. The approach of semi-structured interviews and Thematic Analysis successfully identified a range of views and provided information about the contexts in which these views take shape and about which contexts the views could apply.

## 6. Conclusions

Clients expressed support for the use of incentives in treatment and could see CM influencing earlier and extended engagement in treatment, as well as encouraging clients to make progress in recovery from gambling harm. Objective CM verification of gambling behaviour is also likely to assist therapists and gamblers to better able to assess and manage their gambling and find appropriate incentives to encourage their attendance and commitment to recovery. Clients related their views to their own and others’ journeys of recovery, and this is an important aspect of context to consider when designing and evaluating CM interventions. Concerns about deception and some aspects of CM triggering gambling warrant attention in future research.

## Figures and Tables

**Table 1:** Root codes for clients’ views of contingency management for gambling (more than 2 people contributed to each code).

Group of Codes	Root Codes
Objections	Incentives are not necessary
	Incentives are similar to gambling
Possible negative effects and solutions	Attracting people for the wrong reasons
	Clients arguing or jealous
	Focus on incentives not recovery
	Trigger thoughts of gambling
	Negative impact on therapeutic relationship
	Non-attendance penalised when genuine
	Relapse penalised
	Clients disheartened if goals not achieved
	Use incentives to gamble
Positive views	Achieving goals means seeing progress
	CM linked to positive outcomes
	General positive statements (e.g., “it’s a good idea”).
	Incentives would be appreciated
	Incentives encourage other positive reinforcement
	Incentives are needed
	Incentives are not a moral issue
	Positive impact on the therapeutic relationship
	Incentives could lead to long term change
Barriers and potential solutions	Cost
Limitations	Incentives don’t address the underlying issues
	Incentives are not enough on their own
	Incentives will lead to short term change
Motivation	It might tip the balance
	You need to be ready
	Helpful alongside other motivations
What, which, how, who	Duration of incentives
	How you do it matters
	Schedule preferences
	What to reward
	Which incentives to use
	Will work best for some groups

**Table 2:** Client characteristics.

Characteristic	N	%
**Region**		
Greater London	3	12
East England	3	12
West Midlands	2	8
South England	3	12
North England	6	24
Scotland	1	4
Wales	4	16
Yorkshire/Humber	3	12
**Recovery Stage**		
Help seeking	1	4
Reduced Gambling	1	4
Abstinent <12 months	9	36
Abstinent 12 months–5 years	10	40
Abstinent >5 years	3	12
Abstinent 12 months–5 years with recent relapse	1	4
**Age Group**		
18–24	1	4
25–34	4	16
35–44	7	28
45–54	9	36
55–64	4	16
**Sex**		
Female	6	24
Male	19	76
**Ethnicity**		
Asian or Asian British	1	4
White British	22	88
Other White (including regions of British Isles)	2	8
**Education (for example)**		
No formal qualifications	2	8
Level 1 (e.g., GCSE grade D–G)	1	4
Level 2 (e.g., GCSE grade A*–C)	7	28
Level 3 (e.g., AS/A level)	4	16
Level 4 (e.g., Cert of HE/BTEC)	0	0
Level 5 (e.g., Diploma)	2	8
Level 6 (e.g., Bachelor’s degree)	7	28
Level 7 (e.g., Master’s degree)	2	8
Level 8 (e.g., Doctorate)	0	0
**Relationship Status**		
Married	6	24
Divorced	3	12
Separated	2	8
Co-habiting	2	8
In a relationship	4	16
Single	8	32
**Living with**		
Alone	6	24
Partner and children (under 18 or over 18)	9	36
Partner	3	12
Children (under 18 or over 18)	2	8
Other family	3	12
Other non-family	2	8
**Accommodation**		
Home owned by family or partner	2	8
Home owned by you	10	40
Privately rented	7	28
Rented from local authority or housing association	4	16
Supported lodgings	1	4
Accommodation provided by work	1	4
**Employment status**		
In paid employment or self-employed	19	76
Not working due to long-term illness	3	12
Unemployed and actively seeking work	1	4
In training/education	1	4
Full time carer	1	4
**Monthly income after tax**		
Less than £1000	6	24
Between £1000 and £1500	4	16
Between £1500 and £1900	6	24
Between £1900 and £2300	2	8
Between £2300 and £2750	2	8
Between £2750 and £3160	1	4
Between £3160 and £3580	2	8
Not disclosed	2	8
**Debt**		
No debt	8	32
Less than £1000	4	16
£1000–£5000	5	20
£6000–£10,000	2	4
£11,000–£15,000	1	4
£16,000–£20,000	1	4
>£20,000 (£36,000–£40,000)	1	4
Not disclosed	2	8

**Table 3:** Description of themes.

Theme	Description	Sample Quotes
1. Dishonesty of active addiction disrupting CM.	Some clients can be expected to manipulate the CM intervention Bank statements could be faked Vouchers could be sold for cash	“It’ll be like lip service and they’ll just do that so they can get whatever” (P11). “The whole client-counsellor relationship is all about kind of honesty and people opening up, and I think you could potentially with some people have an element of dishonesty in order to get the reward” (P21).
2. Get people through the door and keep the coming back.	CM could help to bring people into treatment earlier and improve client retention Relieve the suffering associated with ongoing gambling by encouraging people into treatment earlier than might occur naturally CM could put recovering clients in contact with triggers that could lead to relapse	“I see how many people have come into (myself included) [treatment provider redacted] rooms and then not come back....and I think anything that gives them a fighting chance of eventually getting it, of getting it and staying with it”. (P13)
3. Avoidance of triggers in early recovery could be contradicted by CM *Sub-theme*: Avoiding the means to gamble Avoiding activities similar to gambling Avoiding uncomfortable feelings	CM could put recovering clients in contact with triggers that could lead to relapse Collecting rewards through CM could reinforce gambling related behaviours	“It’ll just wake up them horrible thoughts in somebody’s head…. they’re then thinking, you know how they can dodge and weave to make the voucher into some gambling tokens” (P12). “You go to therapy to stop doing all of those things, stop being reliant on knowing that every Tuesday there might be a bonus in your account when you open up… it would be just absolute no, no, really. It’s almost like it’s putting you back into that place that you’re trying to step away from” (P22).
4. CM could ‘spur you on’ in early recovery	Incentives encourage small steps towards change Rewards could be beneficial to abstinence in the early stages of recovery	“I think people would be immensely proud if they got a reward” (P2). “It just gives a bit of validation for your own personal work” (P20).

## Data Availability

The datasets are not available due to the sensitive nature of the study, as they contain confidential information that could compromise participant confidentiality and consent.

## Supplementary Materials

The following supporting information can be downloaded at: https://www.mdpi.com/article/10.3390/ijerph192417101/s1.
