# Supplementary material for: Client Views of Contingency Management in Gambling Treatment: A Thematic Analysis
Source: Int J Environ Res Public Health. 2022 Dec 19;19(24):17101. doi: 10.3390/ijerph192417101 (PMC9778966; doi:10.3390/ijerph192417101)
Supplement: Supplementary file 1 [file ijerph-19-17101-s001.zip › ijerph-1985378-supplementary.pdf]

Supplementary Materials

INTERVIEW TOPIC GUIDE

1. Description of contingency management and follow-on questions (5 minutes)

Aim: to ensure understanding of Contingency Management (CM), and how it might be applied to gambling

a) Provide the below explanation of CM.

Contingency Management (CM) is a behavioural treatment aimed at increasing positive behaviour change. It usually involves giving clients incentives, such as shopping vouchers, when an agreed therapeutic goal or behaviour is met. For example, the target behaviour of attending a treatment intervention would be expected to increase, or the target of abstinence could be maintained, if followed by rewards.

Incentives such as vouchers can be exchanged for food or goods, or credits towards the purchase of an item chosen by the client. Money is never used as a reward.

Goals could include attendance, abstinence, or personal recovery related goals; it is important that there is an objective way to verify that a goal has been attained. Clients know in advance that they can earn these incentives and what they must do get them. Therapeutic goals are broken down into steps so that any one step toward the goal is not too difficult for the client to achieve.

2. Semi-structured interview (20 minutes)

Aim: to gain the participants perspective on the CM approach applied to gambling.

a) In your opinion, do you feel this could be a successful approach in treatment for people who want help for their gambling problem? Please give reasons for your answer.
b) Would you be interested in taking part in such a program if it became available? How do you think you would respond to this type of approach if it was included in your treatment program?
c) What type of barriers to success do you think this type of approach might encounter?
d) Scenario 1: Throughout a 3-month program every time a client shares a bank statement clear of gambling activity (observed twice a week), he or she receives credits that are exchangeable for goods or services. Each credit is worth £1. The first clear statement earns 3 credits. If the client continues to remain abstinent, each subsequent clear bank statement increases the number of vouchers earned by 1 (i.e., second clear in a row= 4 credits, third = 5 credits). A bank statement indicating unexplained expenditure or failure to submit a statement resets the value of the credits to the initial 3-credit position. No cash is given. Staff members assist in the exchange of credits for items. Clients may choose from a wide variety of items. Some examples may include health club memberships, movie passes, gift certificates to local restaurants, etc. Therapists approve only those exchanges that support gambling-free prosocial activities, then someone from the clinic orders or purchases the item. What do you see as the strengths and weaknesses of this approach?
e) Scenario 2: A client presents to a gambling treatment service for the fourth time, asking for help. Each time he or she presented in the past they did not engage for more than two sessions. The client is offered a six-week, weekly group intervention which will include a reward for attendance. Each session attended is rewarded with a voucher that can be exchanged for food or other goods. The first session attended is rewarded with a £5 voucher, the second consecutive attendance increases to a £6 voucher, and so on. If a session is missed, then the reward begins again at a value of £5. What do you see as the strengths and weaknesses of this approach?

3. **CM attitudes and beliefs -structured surveys (see below; 10 minutes)**

Aim: to field test these surveys with client participants, and identify the prevalence of specific beliefs in the study group

Questionnaires will be visible on the shared whiteboard within Zoom or Teams, answers will be given verbally and recorded by the researcher.

a) Client Survey of Incentives
b) Contingency Management Adoption Attitudes

4. **Semi-structured interview continued (15 minutes)**

Aim: to gain the participants perspective on the CM approach applied to gambling.

a) Probe answers to PSI and CMAA which were the most extreme: you rated Q.—as ------, can you say more about what made you decide to rate it this way?

***Provider Survey of Incentives* (PSI; adapted for clients' view of gambling treatment)**

Please use your experience and opinions to honestly respond to the following questions.

	Strongly disagree	*Disagree*	*Neutral*	*Agree*	*Strongly agree*
1. Overall, I would be in favour of adding an incentive program to my treatment program.					
2. If you give a tangible incentive to clients who've earned them, but not to others, it will result in clients arguing about rewards.					
3. Most clients would sell the tangible incentives they receive.					
4. Clients who sell their tangible incentives will use the money to continue their gambling.					
5. Tangible incentives are worthwhile because they can get clients in the door for treatment.					
6. If a client is abstinent just to get the incentive, it could hurt the treatment process.					
7. Many clients will see rewards for abstinence as cheesy or artificial.					
8. Incentives are just not right because they are rewarding the client for what he/she should be doing in the first place.					
9. Overall, incentives are good for the client/counsellor relationship.					
10. Overall, incentives have negative effects on the client/counsellor relationship.					
11. Incentives are more likely to have positive effects on the client than they are to have negative effects.					
12. Incentives are more likely to have negative than they are to have positive effects on clients.					
13. Incentives will cause jealousy among clients who do not get them.					
14. It would not be right to give an incentive for goals such as attendance if they are not proving they are abstinent from gambling.					

15. It would not be right to give an incentive to clients for not gambling when they are not fulfilling other treatment goals, such as attending a group.					
16. Incentives are useful if they reward clients for fulfilling treatment goals other than just providing evidence of not gambling, such as for regular attendance.					
17. The best incentive programs reward clients only when they are fulfilling multiple treatment goals (e.g., attending and abstinent to get a reward).					
18. The best incentive programs reward clients for only one treatment goal at a time (e.g., attending a group meeting).					
19. Incentive programs that require appointments with clients twice a week are not practical because most programs do not offer appointments twice a week.					
20. Incentive programs that require proof of abstinence at least once a week are not practical because most programmes do not routinely do this.					
21. Incentives help the client achieve abstinence from gambling, allowing the counsellor to focus on helping them make other life changes.					
22. Incentive programs are not consistent with my philosophy of treatment.					
23. Incentives will stop the client from seeing beyond the external reward and prevent them from realizing their internal motivation.					
24. Incentives are a bribe.					
25. Abstinence will only last for as long as the incentives are given					
26. Giving incentives for verified abstinence from gambling helps the client to become abstinent.					
27. Giving incentives for treatment attendance will not improve attendance.					
28. An advantage of incentive programs is that they focus on what is good in the client’s behavior (i.e., the ability to become abstinent), not what went wrong in their recovery.					

29. Consistently providing the client with incentives is likely to push the client back into denial					
30. Any source of abstinence motivation, not just internal motivation, is a good thing for treatment.					
31. Incentive programs that require close tracking of client behaviour are too labour intensive to incorporate into our programme.					
32. Incentives are not useful for short-term treatments (e.g., 1 month or less)					
33. There are enough rewards in being abstinent; incentives are not necessary.					
34. Incentives do not address the underlying issues of addiction.					
35. Incentives can be useful whether or not they address the underlying issues of addiction.					

Contingency Management Adoption Attitudes **(CMAA)**

Please use your experience and opinions to honestly respond to the following questions.

	Strongly disagree	*Disagree*	*Neutral*	*Agree*	*Strongly agree*
1. It is okay for patients to have the opportunity to earn prizes worth as much as £100 for abstinence.					
2. It is okay to pay patients for attending treatment.					
3. Incentives can have a positive effect on the patient/counsellor relationship.					
